# Measurement of lipogenic flux by deuterium resolved mass spectrometry

**DOI:** 10.1038/s41467-021-23958-4

**Published:** 2021-06-18

**Authors:** Xiaorong Fu, Stanisław Deja, Justin A. Fletcher, Norma N. Anderson, Monika Mizerska, Gonçalo Vale, Jeffrey D. Browning, Jay D. Horton, Jeffrey G. McDonald, Matthew A. Mitsche, Shawn C. Burgess

**Affiliations:** 1 Center for Human Nutrition, The University of Texas Southwestern Medical Center, Dallas, TX USA; 2 Department of Molecular Genetics, The University of Texas Southwestern Medical Center, Dallas, TX USA; 3 Department of Biochemistry, The University of Texas Southwestern Medical Center, Dallas, TX USA; 4 Department of Clinical Nutrition, The University of Texas Southwestern Medical Center, Dallas, TX USA; 5 Internal Medicine, The University of Texas Southwestern Medical Center, Dallas, TX USA; 6 Department of Pharmacology, The University of Texas Southwestern Medical Center, Dallas, TX USA

**Keywords:** Lipids, Mass spectrometry, Non-alcoholic fatty liver disease

## Abstract

De novo lipogenesis (DNL) is disrupted in a wide range of human disease. Thus, quantification of DNL may provide insight into mechanisms and guide interventions if it can be performed rapidly and noninvasively. DNL flux is commonly measured by ^2^H incorporation into fatty acids following deuterated water (^2^H_2_O) administration. However, the sensitivity of this approach is limited by the natural abundance of ^13^C, which masks detection of ^2^H by mass spectrometry. Here we report that high-resolution Orbitrap gas-chromatography mass-spectrometry resolves ^2^H and ^13^C fatty acid mass isotopomers, allowing DNL to be quantified using lower ^2^H_2_O doses and shorter experimental periods than previously possible. Serial measurements over 24-hrs in mice detects the nocturnal activation of DNL and matches a ^3^H-water method in mice with genetic activation of DNL. Most importantly, DNL is detected in overnight-fasted humans in less than an hour and is responsive to feeding during a 4-h study. Thus, ^2^H specific MS provides the ability to study DNL in settings that are currently impractical.

## Abstract (web summary)

Fat synthesis is necessary for normal physiology, but its dysregulation contributes to the pathology of many diseases. Here, the authors report a high-resolution mass spectrometry approach that quantifies fat synthesis flux in humans and mice following a brief and low dose of deuterated water.

## Introduction

De novo lipogenesis (DNL) is the synthesis of fatty acids from non-lipid nutrients, such as carbohydrates, and is essential for long-term calorie storage and the maintenance of cell membranes. This process occurs in the cytosol of most cells but is highest in liver and adipose tissues^1^. Lipogenic genes are regulated by sterol regulatory element-binding protein-1c (SREBP-1c)^2^ and carbohydrate-responsive element-binding protein (ChREBP)^3^ in response to cell signaling and glycolytic intermediates. Overactivation of these transcription factors during obesity and insulin resistance triggers non-alcoholic fatty liver disease^4^ (NAFLD) by elevating hepatic DNL^5–7^. NAFLD carries additional risk for dyslipidemia, cardiovascular disease, steatohepatitis, cirrhosis, and hepatocellular carcinoma^8^. DNL is also elevated in some types of cancer cells^9^, may be disrupted in central nervous tissue during neurological disease^10^, and plays a role in the transformation of immune cells^11^. In addition to its role in nutrient metabolism, DNL generates endogenous ligands essential for cell signaling pathways such as PPARα^12,13^, and small amounts of DNL in mitochondria are necessary for normal mitochondrial function^14^. Hence, the ability to monitor the function of DNL in settings ranging from organelles, cells, tissue biopsies, mouse models, and humans will facilitate the discovery of new molecular physiology and potential interventions for many different diseases.

**Fig. 1:**
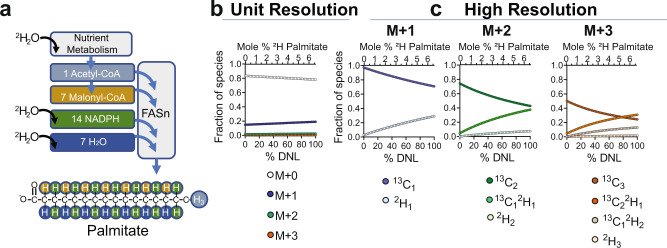
Incorporation of deuterium into palmitate and detection by MS. **a** ^2^H is incorporated into acetyl-CoA during nutrient metabolism and subsequently into palmitate during the reactions of FASn. A newly synthesized palmitate will contain ^2^H from body water, NADPH, malonyl-CoA, and acetyl-CoA. The isotopic enrichment of palmitate measured by GC–MS can be used to quantify the fraction of newly synthesized palmitate. **b** Simulation of unit resolution mass spectra of palmitate in the setting of 0.3% ^2^H body-water enrichment and fractional DNL fluxes of 0–100%. **c** Simulations of the high-resolution mass spectra of palmitate under the same conditions. Simulations are available as Source Data.

As with most metabolic pathways, measuring DNL flux by isotope tracers requires that precursors and products of the pathway be well defined. DNL begins with the carboxylation of acetyl-CoA to malonyl-CoA by acetyl-CoA carboxylase. Fatty acid synthase (FASn) condenses acetyl-CoA and sequential units of malonyl-CoA and, after each elongation, reduces carbonyl groups by NADPH-dependent reductases and H^+^ captured from water. Thus, each 2-carbon unit of a new fatty acid originates from acetyl-CoA, and its hydrogens originate predictably from NADPH, H_2_O, and the original acetyl/malonyl-CoA methyl group (Fig. 1a)^15^. The primary product of FASn is palmitoyl-CoA, though elongase and desaturase enzymes can subsequently produce all non-essential fatty acids. In the liver, new fatty acyl-CoAs are largely converted to triglyceride (TG) and secreted into circulation as VLDL. Thus, palmitate isolated from liver TG or circulating TG is used as an analyte for hepatic DNL measurements.

Carbon isotope (^13^C and ^14^C) labeled substrates that form acetyl-CoA or hydrogen isotopes (^2^H and ^3^H) of body water are candidate tracers of DNL^1,15,16^. Lipogenic ^14^C-substrates that form acetyl-CoA (e.g., glucose, acetate, etc.) detect DNL with high sensitivity, but quantification is complicated since dilution of the lipogenic acetyl-CoA pool is difficult to assess. More sophisticated ^13^C approaches, such as mass isotopomer distribution analysis (MIDA), provide acetyl-CoA dilution and fractional DNL by a statistical analysis of palmitate mass isotopomers^17^. However, the method requires constant infusion to maintain steady-state precursor enrichment, which is impractical for extended experimental periods. Hydrogen isotope tracers of water overcome this limitation because its enrichment diminishes very slowly following initial administration and can be maintained for months with enriched drinking water^6^. When present, hydrogen isotopes of body-water incorporate into new palmitate at known positions and stoichiometry (Fig. 1a). Measuring tritium (^3^H) labeled FAs is sensitive and straightforward^18^, but safety and logistical considerations limit the use of radiolabeled tracers in preclinical models and virtually prohibit their use in humans. In contrast, administration of ^2^H_2_O is safe, inexpensive, convenient, and has been used extensively to estimate DNL in vivo^1^.

Gas chromatography-electron ionization-MS (GC-EI-MS) detects palmitate (as methyl palmitate, C_17_H_34_O_2_) with high sensitivity, and ^2^H incorporation is commonly measured as mass shifts in the spectra (i.e., M + 1, M + 2, M + 3, etc.)^16^. The ^2^H labeling of a new lipid molecule is a function of DNL flux, body-water enrichment, and the total number of exchangeable hydrogens. Palmitate has 31 hydrogens, but only 22 are exchangeable during synthesis^19^. Hence, the ^2^H fractional enrichment of the palmitate pool increases over time towards a maximum enrichment that is proportional to DNL and 22 x body-water enrichment. Typical ^2^H_2_O doses in mice enrich body-water to ~5%^20^, but ~0.5% enrichment is more common in human studies^6,19^. To reach this body-water enrichment, human subjects must drink ~200 mL of 70% ^2^H_2_O, which is usually divided over several hours to avoid nystagmus and nausea^21^. This staggered administration prolongs the study period, limits throughput, and convolutes the first several hours of labeling during an acute ^2^H_2_O study. Here, we investigate the use of high-resolution Orbitrap gas chromatography mass spectrometry (HR-Orbitrap-GC–MS) to resolve ^2^H and ^13^C fatty acid mass isotopomers. We report that this approach allows ^2^H_2_O doses that are 50-times lower than the typical mouse dose and two times lower than the typical human dose, and that tracer incorporation into plasma TG-fatty acids can be detected 1-h after administration. The combination of lower doses and rapid detection will allow DNL flux to be investigated in clinical and basic studies that were previously impractical.

## Results

### ^2^H-palmitate and body water measurement by HR-Orbitrap-GC–MS

The low natural abundance of ^2^H (0.015%) makes tracer doses of less than 0.5% theoretically possible, but quantitation of low ^2^H enrichment by GC–MS is limited by natural abundance isotopes of other nuclei in the analyte^16^. Most notably, the 1.1% natural abundance of ^13^C produces a background M + 1 that is ~18% of the M + 0 palmitate ion. Natural abundance correction algorithms provide reliable ^2^H mass enrichments, if excess enrichment in the mass isotopomer distribution can be detected^16^. Under ideal conditions, this approach is limited to >0.1% molar excess ^2^H in palmitate^19^. A binomial model of ^2^H enrichment^16^ predicts that a fractional DNL flux of 10% in the presence of 0.3% body water will produce 0.7% molar excess ^2^H enriched palmitate, which is consistent with previous DNL measurements in humans under these parameters^19^. However, the challenge of making this measurement is illustrated by simulation data that predicts nearly imperceptible increases in M + 1 (+3%) and M + 2 (+5%) as the fraction of new lipid (fractional DNL) ranges from 0 to 10% (Fig. 1b).

In principle, palmitate ^13^C_1_ and ^2^H_1_ (M + 1) mass shifts can be resolved with a minimum resolving power of 165,000^22^. Baseline separation of ^13^C_1_ and ^2^H_1_ has been demonstrated for palmitate and peptides using Fourier transform ion cyclotron resonance mass spectrometry^23^, an instrument that can achieve a resolution >600,000, but requires long scan times (~4 s), a high-field superconducting magnet, and is generally restricted to MS technology development laboratories. More recently, high-resolution Orbitrap MS detection has become commercially available, making it feasible to resolve ^13^C and ^2^H mass shifts in metabolism labs. To establish whether this approach provides an advantage over unit resolution MS, we simulated ultra-high-resolution MS (HRMS) mass isotopomers of ^2^H-palmitate across a 0–10% range of fractional DNL and a 0.3% body water enrichment. In contrast to unit resolution M + 1 (Fig. 1b), the ^2^H_1_ M + 1, ^2^H_2_ M + 2, and ^2^H_3_ M + 3 signals increase robustly across this range (Fig. 1c). Thus, HRMS provides a major theoretical advantage for detecting DNL at very low precursor and product enrichments.

**Fig. 2:**
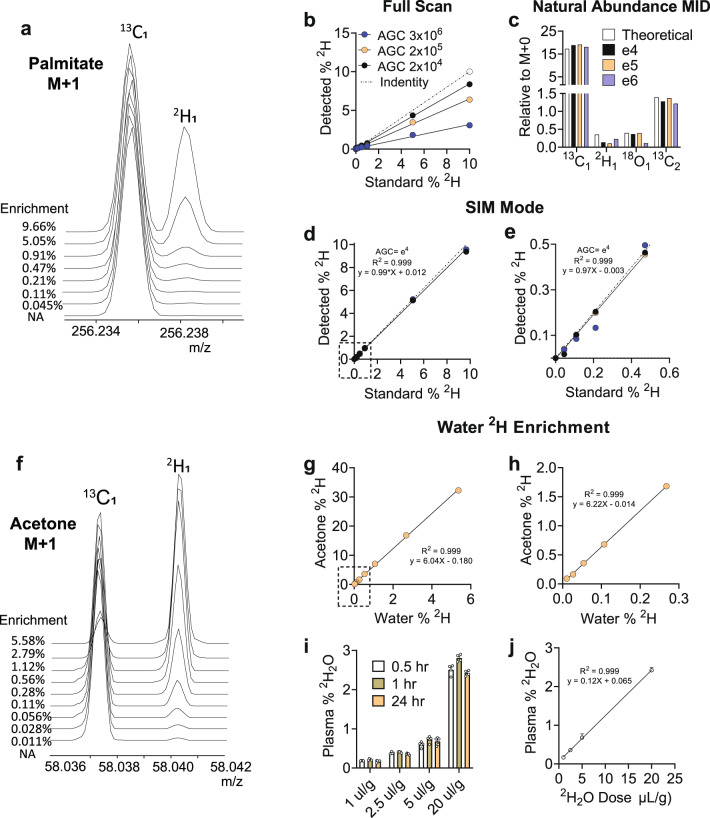
Validation of ^2^H enrichment in palmitate and body water. **a** High-resolution M + 1 mass spectra of [1-^2^H_1_]palmitate enrichment standards. **b** Calibration curve of [1-^2^H_1_]palmitate enrichment standards by full scan acquisition (each point measured in triplicate). **c** Natural abundance MID acquired by t-SIM at different AGC targets. **d** Calibration curve of ^2^H**-**palmitate enrichment standards by t-SIM acquisition at different AGC targets. **e** Expansion of curve for low enrichment samples. **f** High resolution M + 1 mass spectra of ^2^H-acetone enrichment standards generated by using various ^2^H_2_O enrichments. **g** Calibration curve of ^2^H**-**acetone enrichment standards by t-SIM acquisition. **h** Expansion of curve for low enrichment samples. **i** Evolution of plasma body water enrichment with different ^2^H_2_O doses in mice (*n* = 5 mice per dose except for the 20 μl/g dose which had *n* = 4). **j** Plasma ^2^H_2_O enrichment as a function of ^2^H_2_O dose in mice (*n* = 5 mice per dose except for the 20 μl/g dose which had *n* = 4). Where appropriate Mean and SEM are shown and R^2^ determined by simple linear regression.

To experimentally evaluate the approach, we first determined whether ^13^C_1_ and ^2^H_1_ M + 1 in [1-^2^H_1_] palmitate standards are resolved by HR-Orbitrap-GC–MS with 240,000 resolution (FWHM, m/z 200). Pentafluorobenzyl bromide was used to derivatize palmitate prior to analysis in electron capture negative ion (ECNI) mode. ECNI detection of the pentafluorobenzyl ester produced an unfragmented palmitate-ion (C_16_H_3_1O_2_, exact mass 255.2324) with higher efficiency and a 1000-fold higher sensitivity than EI detection of the methyl ester (Supplementary Fig. 1). It was recently noted that Orbitrap detectors develop analytical artifacts when automatic gain control (AGC) target settings saturate the detector with ions, or when scan windows are too large^22^. Thus, we analyzed palmitate standards using full scan and targeted-selected ion monitoring (t-SIM) at different AGC targets (2e^4^, 2e^5^, and 3e^6^). The M + 1 of ^2^H_1_ and ^13^C_1_ of palmitate standards were well-resolved by both methods (illustrated for t-SIM in Fig. 2a). Next, we evaluated the linearity of the various methods in unlabeled and ^2^H_1_-palmitate standards. Small amounts of ^2^H natural abundance were corrected using an unlabeled standard and a matrix algorithm^24^. As previously reported^22^, there was a loss of ions in full scan mode, as indicated by a slope less than 1 (Fig. 2b). Examination of the raw data (Supplementary Fig. 2) confirmed that full scan data are vulnerable to loss of ions, particularly at high AGC target values, consistent with space charge effects. Palmitate ^2^H was detected in t-SIM mode with good accuracy (94-107%) and precision (coefficient of variation <10%) (Supplementary Table 1). The natural abundance distribution of ^13^C, ^2^H, and ^18^O could be detected, but with lower than expected ^2^H abundance (Fig. 2c), which might indicate some ion loss at very low enrichment (Supplementary Fig. 3). Nevertheless, palmitate standards produced a near ideal response (slope = 1), with no AGC target effect (Fig. 2d). Linearity and slope were maintained at enrichments <0.05% palmitate ^2^H (Fig. 2e), which is 20-fold lower than palmitate enrichments detected in humans during a ^2^H_2_O study^25^, and 2-fold lower than the limit of precision for unit resolution MS^19^. Thus, resolving ^2^H and ^13^C isotopologues allow ^2^H enrichment in palmitate to be determined with high sensitivity and precision.

In addition to detecting ^2^H palmitate at low enrichment, quantifying low ^2^H body-water during a ^2^H_2_O experiment presents separate challenges for DNL measurements. Isotope ratio mass spectrometry (IRMS)^26^ and ^2^H NMR^27^ can detect ^2^H in water at, or below, natural abundance, but compared to GC–MS, these specialized instruments are uncommon in metabolism labs. A simplified technique allows ^2^H_2_O enrichment to be measured following base-catalyzed hydrogen exchange into acetone, with subsequent detection of deuterated acetone by GC–MS^28,29^. Again, the sensitivity of this approach is limited by background ^13^C (~3% total) natural abundance^28^. However, HR-Orbitrap-GC–MS easily meets the minimum resolution (18,000) required to resolve ^2^H and ^13^C isotopomers of acetone^22^. The ^2^H of water could be detected as M + 1 ^2^H_1_ acetone at natural abundance levels (Fig. 2f). Standard curves resulted in a stoichiometric slope with excellent linearity at both high enrichment (0–6%) (Fig. 2g) and low enrichment (0–0.3%) ranges (Fig. 2h). To confirm the reliability of body-water ^2^H_2_O detection, mice were injected with 1–20 µL/g body-weight doses of ^2^H_2_O (saline), and 5 µL blood samples were examined. The ^2^H_2_O dose equilibrated with body-water nearly completely in 0.5-h (Fig. 2i), and body-water ^2^H_2_O enrichment was precisely proportional to injection volumes (Fig. 2j). The 1 µL/g ^2^H_2_O dose was easily detected as a ~0.15% body-water enrichment, which is 2 to 3-fold less than the body-water enrichment used in humans. Thus, HR-Orbitrap GC–MS detects both plasma TG/palmitate and body-water at low ^2^H enrichments necessary for in vivo determination of DNL flux.

### Quantifying DNL in mice

**Fig. 3:**
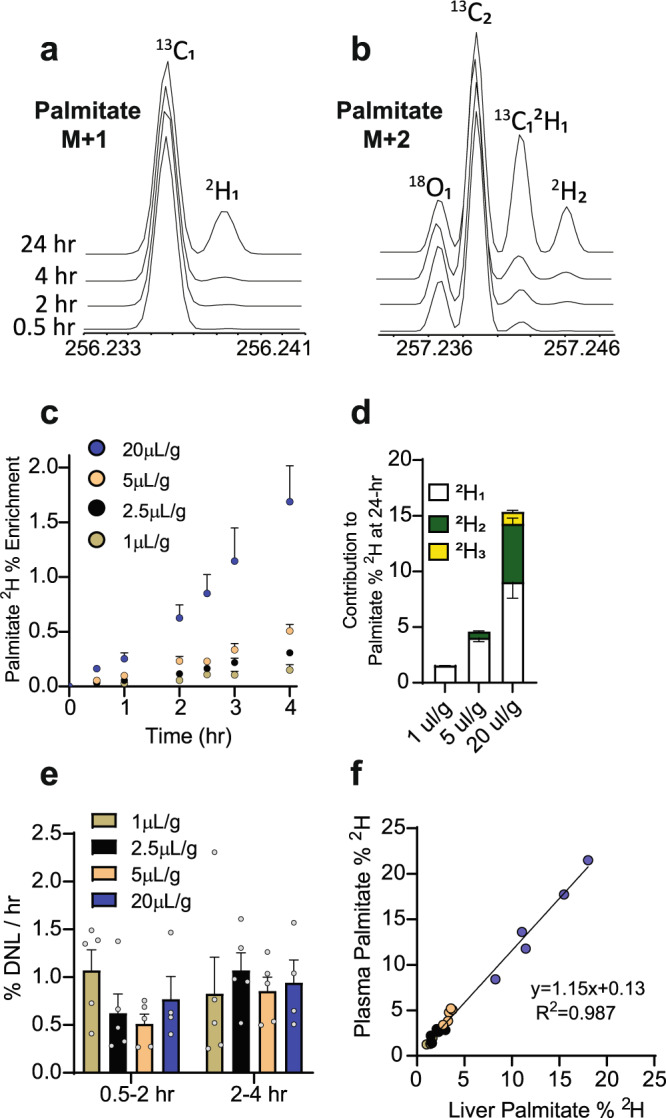
^2^H_2_O administration and serial measurements of lipid synthesis in mice. High resolution (**a**) M + 1 and (**b**) M + 2 mass spectra of ^2^H-palmitate from the plasma TG of a mouse after a 5 µl/g dose of ^2^H_2_O. **c** Palmitate enrichment kinetics measured after different ^2^H_2_O doses (*n* = 5 mice per dose for all doses except the 3-h time point which had *n* = 4). **d** Contributions of ^2^H_1_, ^2^H_2_ and ^2^H_3_ to palmitate enrichment after 24-h at different ^2^H_2_O doses (*n* = 5 mice per dose). Contributions calculated as 1 x ^2^H_1,_ 2 x ^2^H_2_, and 3 x ^2^H_3_ fractional isotopomer distributions, as described in the Methods section. **e** Initial DNL rates were independent of ^2^H_2_O dose (no significant differences detected by 2-way Anova, *n* = 5 mice per dose except for the 20 μl/g dose which had *n* = 4). **f** Correlation between plasma TG palmitate and liver TG palmitate 24-h after various ^2^H_2_O doses (*n* = 5 mice per dose, total of *n* = 20 mice in the correlation). Mean and SEM shown where appropriate.

We next tested whether these ^2^H_2_O doses (1, 2.5, 5, and 20 µL/g body weight) could be used to quantify DNL in mice. Mice were fasted overnight and refed their normal chow diet 2-h before the administration of ^2^H_2_O. Plasma was collected at 0.5,1, 2, 2.5, 3, 4, and 24-h. HR-Orbitrap-GC–MS provided excellent resolution of palmitate ^13^C_1_ and ^2^H_1_ isotopomers of M + 1 (Fig. 3a), and ^13^C_2_, ^13^C_1_^2^H_1_, ^2^H_2,_ and ^18^O_1_ isotopomers of M + 2 (Fig. 3b). ^2^H isotopomers evolved proportionally with dose and duration of exposure (Fig. 3c). Most palmitate ^2^H enrichment arose from ^2^H_1_ at lower doses, but ~30% ^2^H_2_ and 10% ^2^H_3_ contributions were observed at the highest dose (20 µL/g) (Fig. 3d). This distribution will be further shifted to higher ^2^H_x_ at higher doses, in accordance with a binomial distribution^16^. DNL was determined from palmitate enrichment, body-water enrichment (determined by ^2^H exchange into acetone as described above), and the number of exchangeable hydrogens on palmitate. This latter value was estimated to be ~22 from the ratio of fractional ^2^H_1_ (0.091) to ^2^H_2_ (0.027) in palmitate for the 20 µl/g group (Fig. 3d), which was consistent with the previous studies^19^. The ^2^H_2_O dose had no effect on the rate of DNL (Fig. 3e), confirming that larger doses did not alter feeding. Since clinical applications must assume that plasma TG enrichment reflects liver TG enrichment, DNL was measured in TG-palmitate from mouse liver extracts 24-h after ^2^H_2_O administration (Fig. 3f). There was an excellent correlation (*R*^*2*^ = 0.987) between ^2^H palmitate enrichment in plasma and liver TGs, suggesting that TG-palmitate sampled from blood reflects hepatic DNL in mice.

While palmitate is the primary product of FASn, and the most common fatty acid analyte used to determine hepatic DNL, the synthesis of other non-essential fatty acids in plasma TG was also detected (Supplementary Fig. 4). As expected, palmitate, the major product of FASn and stearate (18:0), a single elongation of palmitate, had the highest, M + 1 ^2^H_1_ enrichments. Desaturase products, like palmitoleate (16:1) and oleate (18:1) had roughly half the M + 1 ^2^H_1_ enrichments compared to their saturated counterparts (Supplementary Fig. 4). Although we focus primarily on palmitate as the principal analyte of DNL, other FA species can be detected and may provide insight into downstream processes like elongation and desaturation, which also contribute to the triglyceride pool.

### DNL in response to feeding and genetic activation in mice

**Fig. 4:**
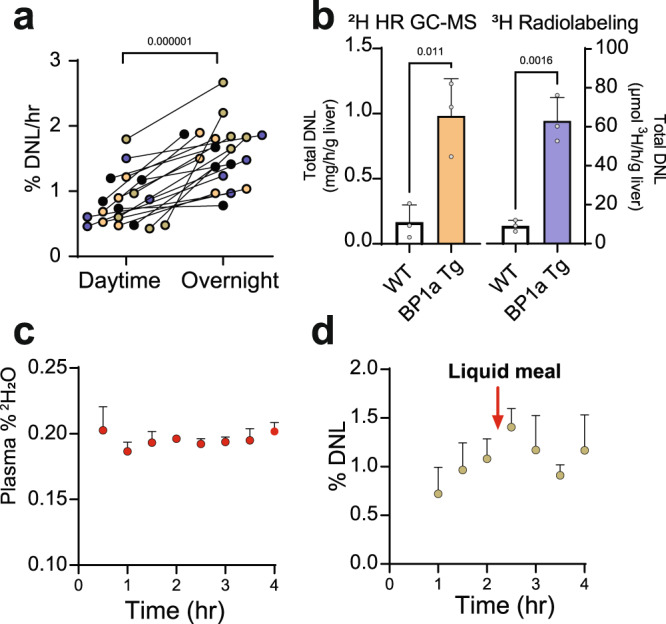
Detection of DNL by high resolution MS is responsive to physiology in mice and humans. **a** Detection of nocturnal feeding in mice at the various doses of ^2^H_2_O (20, 5, 2.5, or 1 μL/g body weight). Colors correspond to the doses as in Fig. 3. DNL in fasted/refed mice was measured in the daytime following a fasting/refeeding protocol (between 9 am and 1 pm) and again in the same mice the next morning. Each point represents an individual mouse administered the indicated ^2^H_2_O dose (*n* = 19 mice). **b** DNL measured by either high resolution detection of ^2^H palmitate or ^3^H radioactivity in hepatic triglycerides of SREBP-1a transgenic mice 1-h after a 500 μl injection of 99.9% ^2^H_2_O (≈20 µl/g) or 50 mCi of ^3^H_2_O (*n* = 3 mice per group). **c** Evolution of deuterium enrichment in plasma TG palmitate after an overnight fast and a ~1 g/Kg body-weight dose of water (*n* = 4 human subjects). **d** Fractional DNL (%) over time following a ^2^H_2_O dose at 0-h and a liquid meal at 2-h (*n* = 4 human subjects). Significance was determined by a two-sided paired (**a**) or unpaired *t*-test (**b**). Mean and SEM are shown where indicated.

To test whether the method detects DNL flux altered by nutritional or genetic interventions, we examined the effect of overnight feeding and genetic activation of DNL in mice. Overnight DNL, regardless of dose, was increased ~2-fold compared to daytime DNL (Fig. 4a), consistent with the expected effect of nocturnal feeding in mice. To induce a more dramatic phenotype, we measured DNL in mice overexpressing a constitutively active form of SREBP-1a^18^. To further confirm the accuracy of the method, liver samples were obtained from mice 1-h after either ^3^H_2_O or ^2^H_2_O administration (Supplementary Fig. 5a), and lipids were analyzed by scintillation counter or HR-Orbitrap-GC–MS. SREBP-1a transgenic mice had 2.5-fold more ^2^H incorporation into palmitate (Supplementary Fig. 5b), 2.4-fold elevated TG-palmitate content (Supplementary Fig. 5c) and an overall 6-fold increase in total DNL flux compared to control mice (Fig. 4b). It is important to note that the total content of ^2^H labeled TG-palmitate measured in this manner is analogous to the ^3^H lipid content measured by scintillation counter and is dependent on both the accuracy of the ^2^H detection and the TG extraction efficiency. Although the extraction approach used here was based on other well-validated methods, there were minor modifications to the solvent ratio and extraction duration which were not systematically evaluated for yield. Nevertheless, DNL measured by HR-Orbitrap-GC–MS closely matched the parallel experiment performed using ^3^H_2_O (Fig. 4b). Thus, DNL flux measurements by HR-Orbitrap-GC–MS can be performed using very low doses of ^2^H_2_O, short experimental times, detects serial changes across nutritional intervals, and provides similar results as ultra-sensitive ^3^H radio-detection.

### Rapid determination of DNL in humans

Finally, we tested whether the method detects DNL flux during a short-term and low dose ^2^H_2_O experiment in humans. Subjects (Supplementary Table 2) consumed an oral dose of ^2^H_2_O at ~1 g/kg body weight after a self-monitored overnight fast. This dose (roughly equal to 75 mL) is 1.5 to 9-fold lower than doses used in other human studies^6,25^ and produced a body water enrichment of ~0.2% (Fig. 4c). The lower dose improves logistics by eliminating divided doses and reduces the occurrence of side effects like nystagmus and nausea^21^. Remarkably, DNL flux was detected in overnight fasted humans 1-h after ^2^H_2_O administration (Fig. 4d). Measuring this flux required detecting 0.03-0.07% mole excess ^2^H palmitate enrichment, which is ~3-fold lower than the detection limit of unit resolution GC–MS^25^. Previous human studies required more than 12-h of ^2^H_2_O exposure and were typically performed after palmitate enrichment reached its zenith^6,25,30^.

To test the responsiveness of DNL, subjects were provided a liquid mixed meal (32 g carbohydrate, 9 g protein, 6 g fat) at the 2-h time-point. This intervention caused a transient decline in fractional DNL, likely due to absorption and esterification of meal fat, and was followed by resumption of palmitate ^2^H labeling 2-h after the meal (Fig. 4d). The lower ^2^H_2_O doses and much shorter protocols allowed by HR-Orbitrap-GC–MS will greatly expand clinical applications of DNL flux measurements.

## Discussion

In this study we introduce a HR-Orbitrap-GC–MS approach, which resolves ^2^H mass enrichment from other isotopes, to study lipogenesis. Direct detection of ^2^H mass isotopomers in palmitate prevents convolution with ^13^C natural abundance at low enrichments. Thus, near-natural abundance ^2^H can be detected, which is not possible using unit resolution MS. DNL could be detected in 1-h and with lower doses of ^2^H_2_O than previously feasible. The dose can likely be lowered another 3-fold, if longer experimental durations are used. Thus, lipogenesis can be evaluated during a short clinical examination, providing the opportunity for large-scale translational studies, or using ultra low doses in vulnerable patient populations. The capacity to collect detailed kinetic data will provide insights into pool sizes and turnover that are lost in a single fractional DNL measurement^6^, particularly if taken outside of steady-state^30^. It is also significant that high-resolution detection allows simultaneous ^13^C and ^2^H tracer experiments without mass interference. We note that new fatty acid incorporation into specific high molecular weight subclasses of lipid esters (i.e. phospholipids and triglycerides, etc.) can be detected by HR-LC-MS or direct infusion analyses with HR accurate MS^31–33^. Recently DNL was determined from the ^2^H enrichment of fatty acyl fragments in the MS/MS spectra of some of these lipid esters, providing insight into the synthesis of specific bioactive lipids^33^. However, the general application of HR-LC-MS to DNL is complicated compared to HR-GC–MS analysis due to the many combinations of fatty acid species and lipid backbones that are detected^32^. While less applicable to large complex lipids, the HR-Orbitrap-GC–MS is simple and generalizable to all FAs, sterols or other metabolites that form fragments below ~300 Da. The ability to measure ^2^H incorporation at very low enrichment without ^13^C interference will allow ^2^H tracer approaches to be used in metabolic studies that were previously impractical.

## Methods

### Animals for kinetic experiments

Animal protocols complied with and were approved by the Institutional Animal Care and Use Committee at the UT Southwestern Medical Center (APN: 2018-102548). The optimal ^2^H_2_O dose and timing for palmitate ^2^H measurements in plasma TG were determined in C57BL/6 J mice purchased from the UT Southwestern Mouse Breeding Core. Mice were maintained on a 12-h/12-h dark/light cycle, in a room with an average temperature of 22 degrees Celsius, 50-70% humidity and unrestricted access to food and water unless otherwise noted. Male mice, 16-18 weeks of age, were fasted 16 h prior to being refed by providing ad libitum access to standard chow (Teklad Diet 2016; Harlan Laboratories). After 2 h of refeeding, mice received IP injections of either 20, 5, 2.5, or 1 μL/g body weight of ^2^H_2_O (*n* = 5 per group). Animals were provided access to ^2^H_2_O labeled drinking water (3, 0.75, 0.375, or 0.15%) corresponding to the expected body-water enrichment estimated from previous studies^20^. Blood was collected from the tail vein at 0, 30, 60, 120, 150, 180, and 240 min after tracer administration. At 24-h post injection, mice were anesthetized with isoflurane, the liver was excised, flash-frozen in liquid nitrogen, and a final blood sample was collected.

### Sample preparation

Plasma samples (5 µL) were treated with 2 mL (1:1, v/v) methanol/dichloromethane (DCM) to precipitate proteins and extract triglycerides, similar to previously reported methods^34,35^. Samples were vortexed for 1 min and then centrifuged for 5 min at 1635 × g. The DCM layer was transferred to a new tube and dried under N_2_. Samples were saponified with 1 mL 0.5 M KOH in methanol at 80 °C for 1 hr. Resulting FAs were extracted with 2 mL DCM/water (1:1, v/v) for 1 min, and the solvent was evaporated under N_2_. Dried lipid extract was resuspended in 50 µL of 1% triethylamine/acetone and reacted with 50 µL of 1% PFBBr/acetone for 30 min at room temperature. To this solution, 1 mL of isooctane was added before MS analysis.

Approximately ~20 mg of the liver was weighed and homogenized with 1 mL methanol/DCM (1:1, v/v) for 1 min in 2.0-mL pre-filled Bead Ruptor Tubes (2.8 mm ceramic beads, Omni International, Kennesaw, GA, USA). The tubes were washed twice with 1 mL methanol/DCM and all solutions were combined. In a new tube, a known amount of [U-^13^C] palmitate was added to the equivalent of 1 mg of liver extract for concentration assays when applicable. Samples were dried under N_2_ before saponification, fatty acid extraction, and derivatization were performed as described above for plasma samples.

### Analysis of samples by HR-Orbitrap-GC–MS

The ^2^H‐enrichment of palmitate (M + 0, M + 1, M + 2, M + 3 mass isotopomers) was determined using a Q Exactive GC-Orbitrap MS (Thermo Scientific). A 1 μL sample was injected onto a HP-5ms capillary column (60 m × 0.32 mm i.d., 0.25 µm film thickness) in split mode. The helium gas flow rate was 1 mL/min for 13 min, followed by 0.4 mL/min for 3 min and then 1 mL/min for the remainder of the run. The GC injector temperature was 250 °C and the transfer line was 290 °C. The column temperature program was 150 °C for 1 min, followed by a 30 °C/min ramp to 250 °C for 12 min, and finally 5 min at 310 °C. Samples were analyzed at 70 eV in ECNI mode with methane gas at 1.5 mL/min. Full scan and t-SIM methods were investigated at different AGC target values (2e^4^, 2e^5^, and 3e^6^) at 240,000 (FWHM, m/z 200) resolution to determine the accuracy and repeatability of palmitate enrichment measurements. In full scan, a scan range of m/z of 250-265 was selected. In t-SIM method, the quadrupole was set to pass ions between m/z 254.2 and 260.2. For both methods, the maximum injection time was 54 ms.

### Enrichment determination

^2^H-mol fraction excess standards for validation experiments were prepared by mixing weighed samples of unlabeled and 99.9% enriched ^2^H_1_-palmitate. Biologically labeled palmitate ^2^H enrichment was determined from mass isotopomers M + 1 (^2^H_1_), M + 2 (^2^H_2_) and M + 3 (^2^H_3_). A small correction for ^2^H_1_ natural abundance was made using the MID of a biological sample (collected before ^2^H_2_O administration) and a matrix correction algorithm^24^. Palmitate ^2^H enrichment = ^2^H_1_ + (^2^H_2_ x 2) + (^2^H_3_ x 3). Higher mass isotopomers may also contribute to body water enrichments above 5% (Fig. 3d). Each sample was measured in triplicate and the average was used for the value of the sample.

Palmitate concentrations were calculated from the ratio of peak areas corresponding to palmitate (m/z 255) and to [U-^13^C] palmitate internal standard (*m*/*z* 271 and 270); standard curves containing known concentration ratios of unlabeled and internal standards were run with the samples.

### Methyl palmitate analysis by GC–MS

Methyl palmitate was prepared as previously described^36^ for direct comparison to the palmitate pentafluorobenzyl ester derivative (Supplementary Fig. 1) used for the analysis described above, using the same instrument. Briefly, palmitate was derivatized to the methyl ester in H_2_SO_4_/methanol at 100 °C for 2 hr. The derivatives were then extracted with a hexane-water mixture. The GC oven temperature was programmed to ramp from 100 °C (isothermal for 1 min) to 250 °C at a rate of 20 °C/min, then to 275 °C at a rate of 10 °C/min. The final temperature was held for 3 min. Helium was used as the carrier gas at a flow rate of 1 mL/min. GC–MS analysis, with 70 eV EI, was carried out in full-scan acquisition mode over a m/z range of 100–300 at 15,000 mass resolution.

### Simulations

High resolution data was simulated as a binomial distribution of ^2^H species dependent on body water enrichment, the number of exchangeable hydrogens and the theoretical natural abundance of ^13^C and ^2^H as a function of %DNL. A spreadsheet with the calculations shown in Fig. 1b, c is provided as Source Data.

### Body water enrichment measurement

Plasma and acetone were incubated under alkaline conditions as previously reported^28,29^. Briefly, 5 μL of plasma, 2 μL of 10 M sodium hydroxide, and 5 μL of acetone were added to a GC vial with a PTFE/silicone threaded cap. Samples were incubated overnight at room temperature prior to analysis. Calibration standards of known ^2^H-mol fraction excess were prepared by mixing weighed samples of naturally labeled and of 99.9% ^2^H_2_O.

Negative chemical ionization mode (NCI) was used with t-SIM acquisition (m/z 55.5-60.5) and 60,000 mass resolution (FWHM, m/z 200) on the same Q Exactive GC-Orbitrap MS instrument as described above. 1 μL of headspace acetone gas from the GC vial was injected in split mode. The column temperature program was 2 min at 60 °C, increased by 30 °C/min to 200 °C for 2 min. The transfer line temperature was 250 °C leading into the ion source (250 °C), which was operated with a methane reaction gas at 1.5 mL/min. The Orbitrap AGC target was set to 5e^4^ with a maximum injection time of 54 ms.

### Data extraction from HRMS MS/MS spectra

Thermo Scientific Xcalibur (version 4.2.47) Data System was used for data collection. Extraction of individual high-resolution *m/z* values representing each isotopomer ion (palmitate and acetone) was performed using TraceFinder 4.1 (Thermo Scientific) with 4 ppm mass tolerance.

### DNL calculations

The contribution of DNL to the pool of total circulating palmitate was determined as previously described^25^. Briefly, ^2^H_2_O quickly equilibrates with the total body water pool, DNL is measured based on the incorporation of ^2^H into palmitate following the administration of ^2^H_2_O. The contribution of palmitate synthesis was determined using Eq. 1:

1 $${DNL}=\frac{{palmitate}\,{enrichment}}{({BW}\times n)}\times 100$$

where DNL is the fraction of palmitate that was newly made since the administration of ^2^H_2_O, palmitate enrichment is expressed as described above, BW (body water) is the fraction ^2^H_2_O in plasma, and “*n*” represents the number of exchangeable hydrogens. The value for *n* was measured to be 22 using ^2^H_1_ and ^2^H_2_ enrichments and Eq. 2 in 24-h plasma samples as previously reported^19^.

2 $$\frac{{\rm{m}}2}{{\rm{m}}1}=\frac{\left(n-1\right)}{2}\times \frac{{BW}}{1-{BW}}$$

DNL/hr is determined by dividing by the time since the administration of ^2^H_2_O. Absolute palmitate synthesis rates in the liver were determined by multiplying DNL by tissue TG-palmitate concentration where noted.

### Comparison of ^2^H_2_O and ^3^H_2_O DNL measurements

Validation of DNL measurements by ^2^H_2_O and ^3^H_2_O was performed using mice that express a transgenic, constitutively active form of SREBP-1a (*n* = 3) and littermate controls (*n* = 3), generated as previously described^18^. 11- to 12-week-old mice were maintained ad libitum on Teklad 2018 chow diet. Food was removed 3-h before mice were injected intraperitoneally with 500 µL of either 50 mCi of ^3^H_2_O or 99.9% of ^2^H_2_O. After 1-h, mice were anesthetized for plasma and tissue collection. Hepatic DNL measurements by ^2^H_2_O were performed as described above. To compare these results to DNL measured using the ^3^H_2_O method^18^, fractional DNL was multiplied by the total amount of liver TG content.

### Human DNL measurements

Human subjects (*n* = 4, Supplementary Table 2) were studied after approval by the UT Southwestern Institutional Review Board. All participants were provided written informed consent before participation. Healthy volunteers were studied after a self-managed overnight fast. A baseline blood sample was drawn prior to ^2^H_2_O administration. Subjects consumed a single oral dose of 70% ^2^H_2_O at 2 g/kg body-water (where body-water was estimated to be 0.6 x body weight in men and 0.5 x body-weight in women), or roughly ~1 g/Kg body weight. Blood samples were collected at 0.5, 1, 1.5, and 2-h. Following the 2-h collection, subjects consumed a liquid meal consisting of 8 ounces of Ensure®, which contains 220 Kcal, 6 g fat, 32 g carbohydrates, 15 g sugar and 9 g protein (Abbot Labs, Columbus OH). Blood collections continued at 2.5, 3, 3.5, and 4-h.

### Statistics, reproducibility, and study design

Results are expressed as means ± standard errors of the mean (SEM). Sample sizes were chosen based on previous experience but without power analysis. All MS data were collected in duplicate or triplicate. Analysis was performed without blinding. Replication of analytical performance is expressed in Supplemental Table 1 as accuracy and precision and was also observed by proportional responses to multiple doses of ^2^H_2_O in mice. Significant differences (*p* < 0.05) were determined using a two-tailed *t*-test. Significant correlations were determined using a two-tailed Pearson correlation. All statistics and graph preparation were performed in Prism 8 (Graph Pad Software Inc.).

### Reporting Summary

Further information on research design is available in the Nature Research Reporting Summary linked to this article.

## Supplementary information

Supplementary Information

Reporting Summary

## Data Availability

The source data underlying each figure and table are provided as a Source Data file. Flux data has been uploaded to Kimosys.org access Data EntryID 129 (mouse) and 130 (human). Source data are provided with this paper.

## Source data

Source data
