# Supplementary material for: Measurement of lipogenic flux by deuterium resolved mass spectrometry
Source: Nat Commun. 2021 Jun 18;12:3756. doi: 10.1038/s41467-021-23958-4 (PMC8213799; doi:10.1038/s41467-021-23958-4)
Supplement: Supplementary file 1 — Supplementary Information [file 41467_2021_23958_MOESM1_ESM.pdf]

Supplementary Information

Measurement of Lipogenic Flux by Deuterium Resolved Mass Spectrometry

Xiaorong Fu[1,2], Stanisław Deja[1,3], Justin A. Fletcher[1,4], Norma N. Anderson[1,2], Monika Mizerska[1], Gonçalo Vale[1], Jeffrey D Browning[4,5], Jay D. Horton[1,5], Jeffrey G. McDonald[1,2], Matthew A. Mitsche[1,2] and Shawn C. Burgess[1,6*]

[1] Center for Human Nutrition, [2] Department of Molecular Genetics, [3] Department of Biochemistry, [4] Department of Clinical Nutrition, [5]Internal Medicine, [6]Department of Pharmacology,

The University of Texas Southwestern Medical Center, Dallas, TX 75390, USA.

***email: shawn.burgess@utsouthwestern.edu**

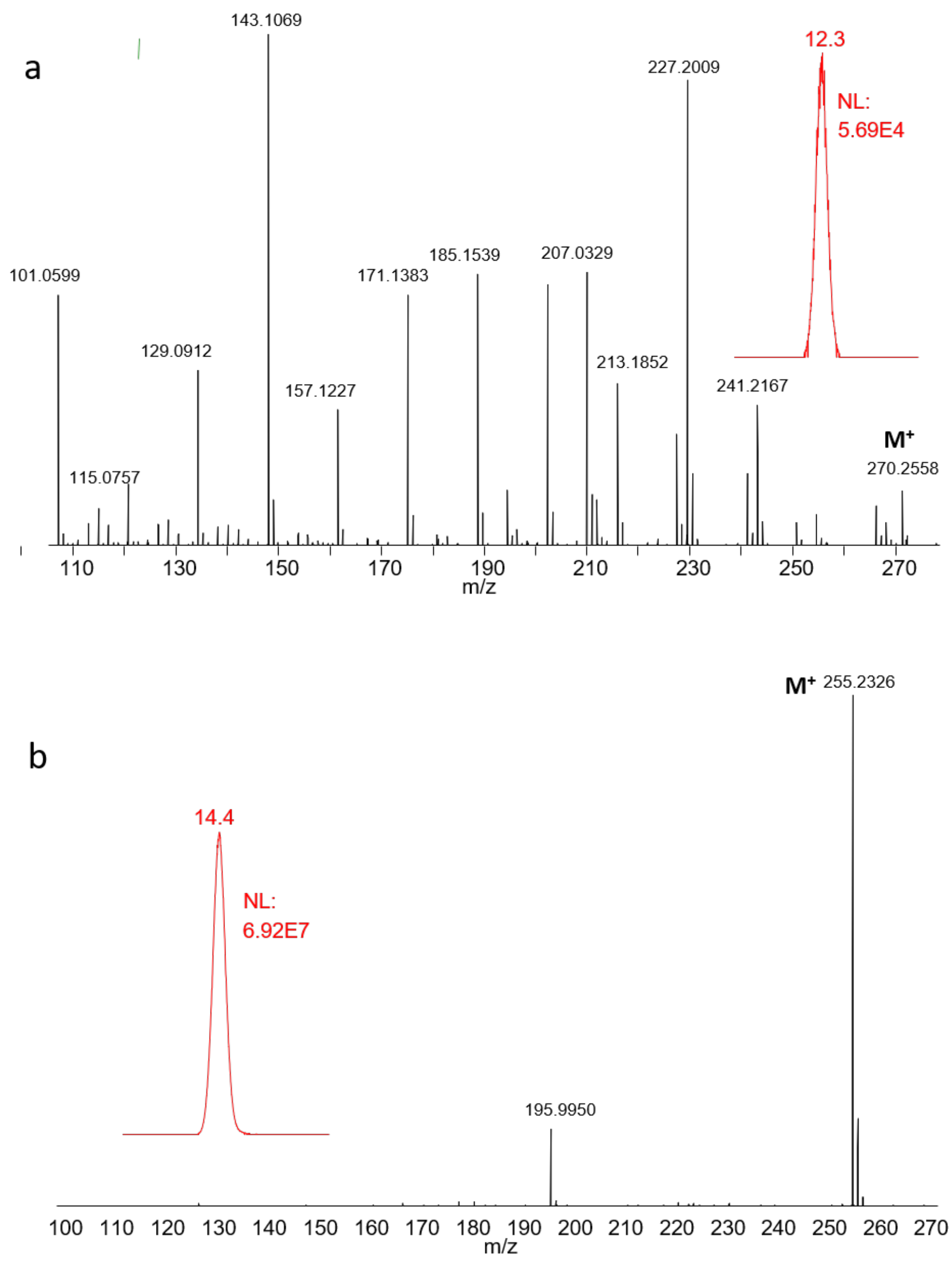

Supplementary Figure 1. Mass spectrum of palmitate derivatives. a Palmitate methyl ester in electron ionization mode **b** Palmitate pentafluorobenzyl ester in ECNI mode. Compared to EI mode, ECNI produced a simpler mass spectrum with a largely intact pseudo-molecular ion. This results in a substantial improvement in s/n compared to the highly fragmented EI spectrum of the methylester, further enhancing the detectability of isotope signatures at very low levels of enrichment. The extracted ion chromatography signal (shown in red) from ion 255.2326 is a thousand times higher than from ion 270.2559.

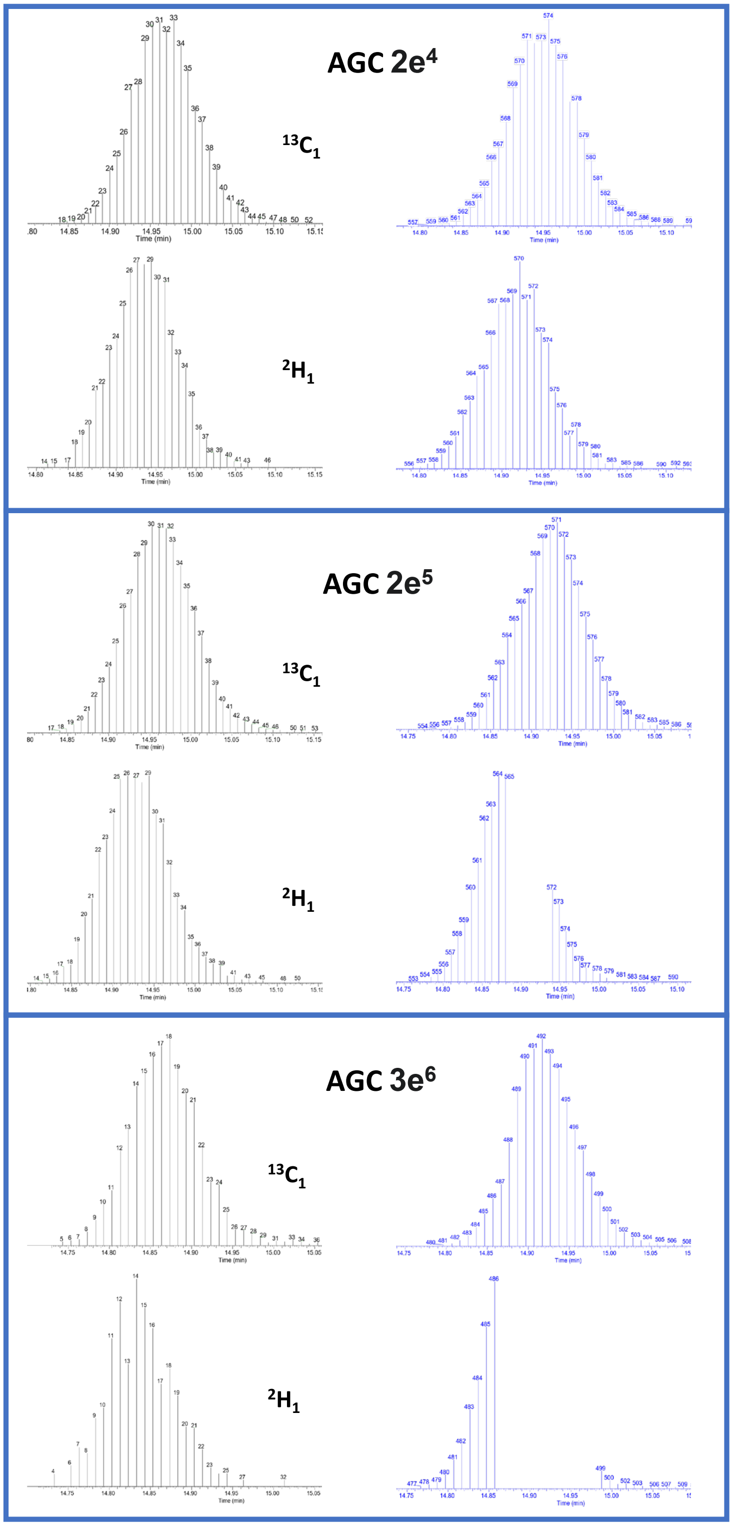

Supplementary Figure 2. Observation of coalescence in full scan acquisition (blue) compared with t-SIM (black) at different AGC values. 5% ^{2}H palmitate enrichment is shown as an example.

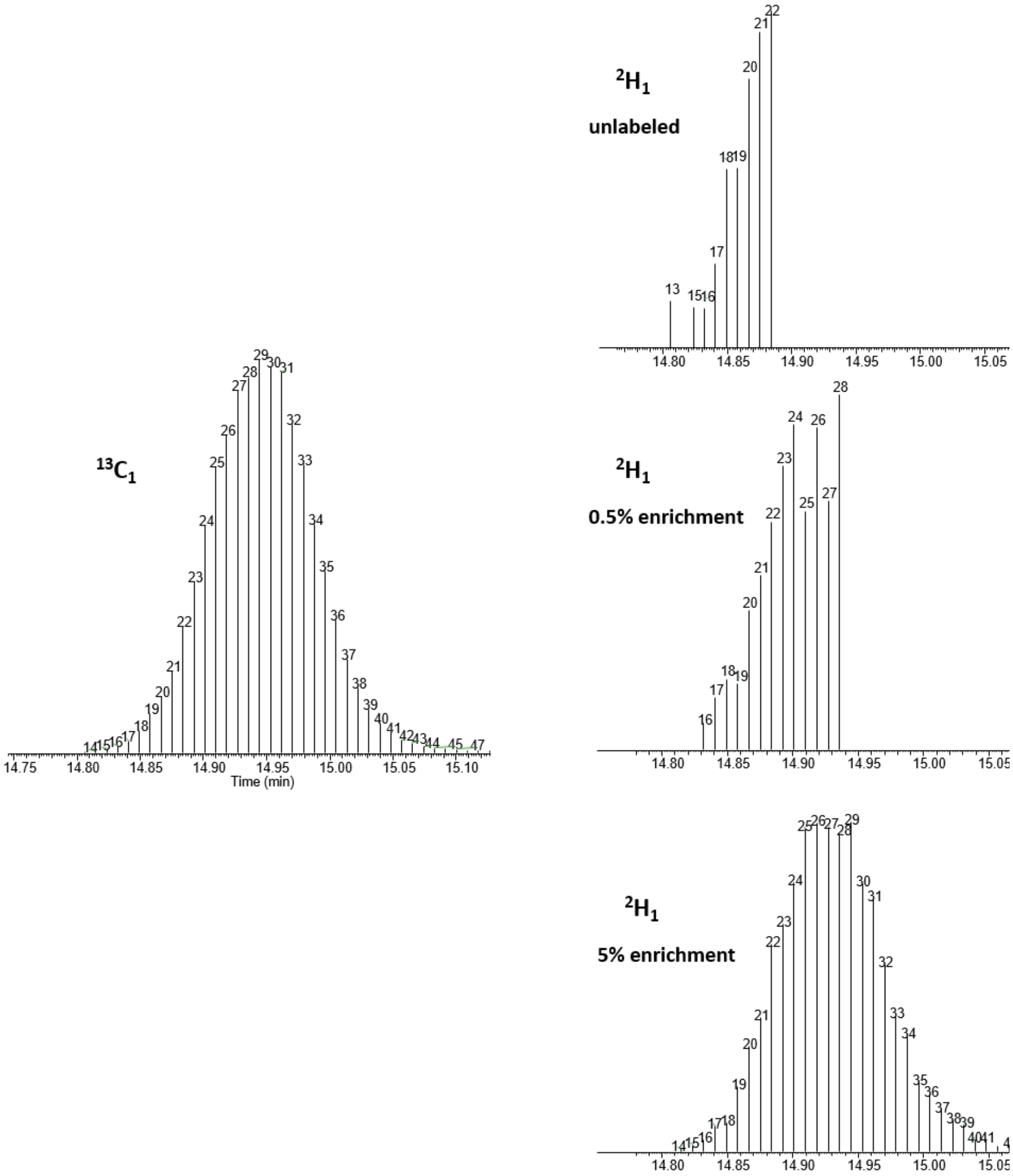

Supplementary Figure 3. Observation of coalescence in t-SIM acquisition for low ^{2}H-palmitate enrichment.

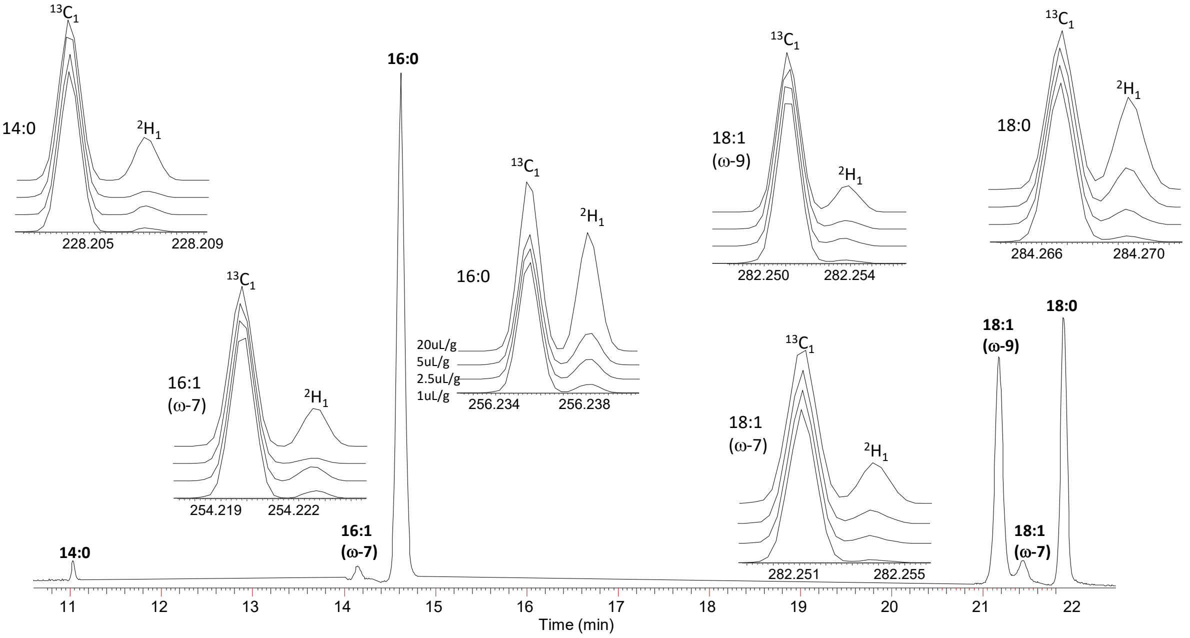

Supplementary Figure 4. GC-MS chromatogram of FAs in mouse plasma. Insets show the M+1 mass spectrum of ^2H labeled FAs after 24-hours at different ^2H_2O dose.

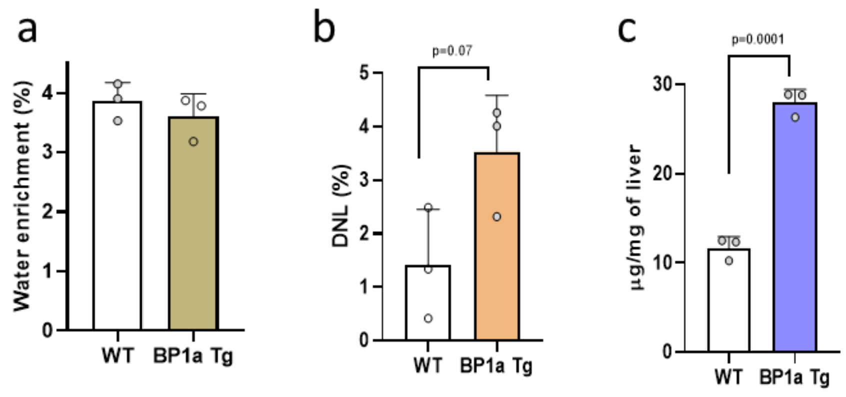

Supplementary Figure 5. Comparison study of $^{2}H_2O$ vs. $^{3}H_2O$ measurements of DNL in mice. a Body water enrichment. **b** DNL and **c** liver TG-palmitate concentration of WT and SREBP-1a mice measured by HRMS. Tissue collected 1-hours after a 500 µL injection of 99.9% $^{2}H_2O$ (approximately 20µL/g) (n=3 mice per group). Results are shown as the mean and SEM. Significance determined by a two-sided unpaired t-test.

Supplementary Table 1. Accuracy and precision of $^{2}H_{1}$-palmitate enrichment measurements by t-SIM

Enrichment by wt (%)	Enrichment by MS (%)	Accuracy (%)	CV (%)
0.05	0.02	66.9	5.5
0.11	0.10	94.4	8.3
0.21	0.20	96.8	11.8
0.47	0.46	98.4	7.6
0.91	0.97	106.5	4.5
5.06	5.13	101.5	1.8
9.66	9.38	97.2	0.2

Supplementary Table 2. Characteristics of human subjects

Age (yrs)	Sex (F/M)	Weight (Kg)	Height (cm)
30 +/- 2.8	1/3	91.5 +/-9.38	177 +/- 4.3

N=4; Mean +/- SEM.
