# Supplementary material for: Measurement of lipogenic flux by deuterium resolved mass spectrometry
Source: Nat Commun. 2021 Jun 18;12:3756. doi: 10.1038/s41467-021-23958-4 (PMC8213799; doi:10.1038/s41467-021-23958-4)
Supplement: Supplementary file 2 — Reporting Summary [file 41467_2021_23958_MOESM2_ESM.pdf]

nature research

Corresponding author(s): Shawn C.Burgess

Last updated by author(s): 04/30/2021

Reporting Summary

Nature Research wishes to improve the reproducibility of the work that we publish. This form provides structure for consistency and transparency in reporting. For further information on Nature Research policies, see our Editorial Policies and the Editorial Policy Checklist.

Statistics

For all statistical analyses, confirm that the following items are present in the figure legend, table legend, main text, or Methods section.

n/a	Confirmed	
☐	☒	The exact sample size (n) for each experimental group/condition, given as a discrete number and unit of measurement
☐	☒	A statement on whether measurements were taken from distinct samples or whether the same sample was measured repeatedly
☐	☒	The statistical test(s) used AND whether they are one- or two-sided<br>*Only common tests should be described solely by name; describe more complex techniques in the Methods section.*
☒	☐	A description of all covariates tested
☐	☒	A description of any assumptions or corrections, such as tests of normality and adjustment for multiple comparisons
☐	☒	A full description of the statistical parameters including central tendency (e.g. means) or other basic estimates (e.g. regression coefficient) AND variation (e.g. standard deviation) or associated estimates of uncertainty (e.g. confidence intervals)
☐	☒	For null hypothesis testing, the test statistic (e.g. F, t, r) with confidence intervals, effect sizes, degrees of freedom and P value noted<br>*Give P values as exact values whenever suitable.*
☒	☐	For Bayesian analysis, information on the choice of priors and Markov chain Monte Carlo settings
☒	☐	For hierarchical and complex designs, identification of the appropriate level for tests and full reporting of outcomes
☐	☒	Estimates of effect sizes (e.g. Cohen's d, Pearson's r), indicating how they were calculated

Our web collection on statistics for biologists contains articles on many of the points above.

Software and code

Policy information about availability of computer code

Data collection	Thermo Scientific Xcalibur (version 4.2.47) Data System was used for high-resolution Orbitrap GC-MS data collection.
Data analysis	Extraction of individual high-resolution m/z values representing each isotopomer ion (palmitate and acetone) was performed using TraceFinder 4.1 (Thermo Scientific).<br>All statistics and graph preparation were performed in Microsoft Excel 365 and Prism 8 (Graph Pad Software Inc.).

For manuscripts utilizing custom algorithms or software that are central to the research but not yet described in published literature, software must be made available to editors and reviewers. We strongly encourage code deposition in a community repository (e.g. GitHub). See the Nature Research guidelines for submitting code & software for further information.

Data

Policy information about availability of data

All manuscripts must include a data availability statement. This statement should provide the following information, where applicable:

- Accession codes, unique identifiers, or web links for publicly available datasets
- A list of figures that have associated raw data
- A description of any restrictions on data availability

All source data underlying the graphs presented in the main figures are reported in the Source Data File. Simulations are available in the Source Data File. Flux data has been uploaded to Kimosys.org access IDs 129 (mouse) and 130 (human). All other data produced by this study are available from the corresponding author upon request.

Field-specific reporting

Please select the one below that is the best fit for your research. If you are not sure, read the appropriate sections before making your selection.

☒ Life sciences ☐ Behavioural & social sciences ☐ Ecological, evolutionary & environmental sciences

For a reference copy of the document with all sections, see nature.com/documents/nr-reporting-summary-flat.pdf

Life sciences study design

All studies must disclose on these points even when the disclosure is negative.

Sample size

Sample sizes of n=4-5 (exact n values given in the figure legends) for mice and n=4 in humans were chosen based on experience with the methodology but without evaluating statistical power. Sample size was consistent with other studies in this field (Ref. 23 and Ref. 25)

Data exclusions

No data were excluded.

Replication

Replication is expressed in Supplemental Table 2 as accuracy and precision (measured three times) and was also observed by proportional responses to multiple doses of 2H2O in mice.

To ensure robust reproducibility, standard curves containing known concentration ratios of unlabeled and labeled palmitate were run every time with the samples to get the accurate enrichment.

Unlabeled biological sample (collected before 2H2O administration) was analyzed at least three times in order to correct the natural abundance of palmitate and acetone.

Comparisons between groups were planned before statistical testing and target effects were not predetermined. Error bars displayed on graphs represent the mean ± SEM of at least three to five independent subjects.

Randomization

Only control human subjects were studied so no randomization was performed.

C57BL/6J mice were randomly assigned in four group and received IP injections of either 20, 5, 2.5, or 1 µL/g body weight of 2H2O (n=5 per group) to quantify DNL in mice.

DNL measurements by 2H2O and 3H2O, SREBP-1a (n = 3) and littermate controls (n = 3) were randomly assigned for either 50 mCi of 3H2O or 99.9% of 2H2O injection.

Blinding

Only control human subjects were examined, so blinding was not applicable to this portion of the study. Validation and method development in mouse studies could not be blinded since multiple instrument settings needed to be tested for accuracy (i.e.AGC targets). There was only one group for the 3H SREBP-1a comparison study, which could not be blinded.

Reporting for specific materials, systems and methods

We require information from authors about some types of materials, experimental systems and methods used in many studies. Here, indicate whether each material, system or method listed is relevant to your study. If you are not sure if a list item applies to your research, read the appropriate section before selecting a response.

Materials & experimental systems

n/a	Involved in the study	
☒	☐	Antibodies
☒	☐	Eukaryotic cell lines
☒	☐	Palaeontology and archaeology
☐	☒	Animals and other organisms
☐	☒	Human research participants
☒	☐	Clinical data
☒	☐	Dual use research of concern

Methods

n/a	Involved in the study	
☒	☐	ChIP-seq
☒	☐	Flow cytometry
☒	☐	MRI-based neuroimaging

Animals and other organisms

Policy information about studies involving animals; ARRIVE guidelines recommended for reporting animal research

Laboratory animals

Animal protocols complied with and were approved by the Institutional Animal Care and Use Committee at the UT Southwestern Medical Center (APN: 2018-102548). The optimal 2H2O dose and timing for palmitate 2H measurements in plasma TG were determined in C57BL/6J mice purchased from the UT Southwestern Mouse Breeding Core. Mice were maintained on a 12-h/12-h dark/light cycle, with unrestricted access to food and water unless otherwise noted. Male mice, 16-18 weeks of age, were fasted 16

	hours prior to being refed by providing ad libitum access to standard chow (NCD; Teklad Diet 2016; Harlan Laboratories). Housing temperature: 72 F; Housing humidity 50-70%
Wild animals	The study did not involve wild animals
Field-collected samples	The study did not involve samples collected from the field.
Ethics oversight	Animal protocols were approved by the UT Southwestern IACUC.

Note that full information on the approval of the study protocol must also be provided in the manuscript.

Human research participants

Policy information about studies involving human research participants

Population characteristics	Subjects (4 normal healthy volunteers) consisted of 3 males and 1 female. Subjects being treated for chronic health conditions were excluded. 3 subjects identified as non-hispanic white and one subject identified as mixed race (black/white). Additional Subject characteristics are provided in supplementary table 2.
Recruitment	Volunteers were recruited by advertisement in public areas of the UT Southwestern Medical Center and by word of mouth. Volunteers were therefore more likely to be individuals working in health care and scientific fields, which might introduce lifestyle bias compared to the general population. The risk of effect on the outcome of the study is minimal, inasmuch as the study was designed to test the sensitivity of a novel approach on healthy control subjects.
Ethics oversight	Study was approved by the UT Southwestern IRB

Note that full information on the approval of the study protocol must also be provided in the manuscript.
